# ‘Thank goodness you’re here’. Exploring the impact on patients, family carers and staff of enhanced 7-day specialist palliative care services: A mixed methods study

**DOI:** 10.1177/02692163231201486

**Published:** 2023-09-21

**Authors:** Catherine Walshe, Céu Mateus, Sandra Varey, Steven Dodd, Zoe Cockshott, Luís Filipe, Sarah G Brearley

**Affiliations:** 1 International Observatory on End of Life Care, Division of Health Research, Lancaster University, Lancaster, England, UK; 2 Health Economics at Lancaster, Division of Health Research, Lancaster University, Lancaster, England, UK

**Keywords:** Palliative care, after-hours care, hospitalisation, health care facilities, manpower and services, out-of-hours medical care

## Abstract

**Background:**

Healthcare usage patterns change for people with life limiting illness as death approaches, with increasing use of out-of-hours services. How best to provide care out of hours is unclear.

**Aim:**

To evaluate the effectiveness and effect of enhancements to 7-day specialist palliative care services, and to explore a range of perspectives on these enhanced services.

**Design:**

An exploratory longitudinal mixed-methods convergent design. This incorporated a quasi-experimental uncontrolled pre-post study using routine data, followed by semi-structured interviews with patients, family carers and health care professionals.

**Setting/participants:**

Data were collected within specialist palliative care services across two UK localities between 2018 and 2020. Routine data from 5601 unique individuals were analysed, with post-intervention interview data from patients (*n* = 19), family carers (*n* = 23) and health care professionals (*n* = 33; *n* = 33 time 1, *n* = 20 time 2).

**Results:**

The mean age of people receiving care was 73 years, predominantly white (90%) and with cancer (42%). There were trends for those in the intervention (enhanced care) period to stay in hospital 0.16 days fewer, but be hospitalised 2.67 more times. Females stayed almost 3.5 more days in the hospital, but were admitted 2.48 fewer times. People with cancer had shorter hospitalisations (4 days fewer), and had two fewer admission episodes. Themes from the qualitative data included responsiveness (of the service); reassurance; relationships; reciprocity (between patients, family carers and staff) and retention (of service staff).

**Conclusions:**

Enhanced seven-day services provide high quality integrated palliative care, with positive experiences for patients, carers and staff.

**What is already known about the topic?**

- Patterns of health care usage appear to change over time, with increasing use of out-of-hours services, such as emergency departments, as death approaches.
- A higher proportion of patients are admitted to hospitals as emergencies on weekends rather than week days.

**What this paper adds**

- Enhanced 7-day specialist palliative care services were associated with trends for shorter but more frequent hospital stays.
- Post initiation of the enhanced 7-day specialist palliative care services, patients and families valued service responsiveness, reassurance and relationships with staff.
- The enhanced service appeared to promote reciprocity between patients, family carers and staff and retention of service staff.

**Implications for practice, theory or policy**

- Seven-day services should continue to be developed or enhanced to provide high quality and integrated palliative care to patients and families.
- If enhanced services are intended to improve access by under-served populations then it is important that this is clearly articulated and the enhancements carefully designed to facilitate this.

## Background

People with life limiting illness account disproportionately for health care utilisation.^1^ Patterns of health care usage appear to change over time, with increasing use of out-of-hours services, such as emergency departments, as death approaches.^2,3^ Because of this, providing equivalent health services on weekends and public holidays to those provided on weekdays has been given increasing international focus, with some guidance indicating that specialist palliative care should be sufficiently staffed to enable face-to-face assessment during normal hours, 7 days a week.^4^ Given the context in most healthcare services of limited resources, it is important that any change to services or their staffing is properly assessed, including an appraisal of cost effectiveness.

Internationally, there is a lack of clarity about the impact of 7-day working on care outcomes. Research into weekend hospital admissions found that a higher proportion of patients were admitted as emergencies on Saturday (50% more) and Sunday (65% more) than week days, and included a greater prevalence of patients with higher mortality risk.^5^ Other research found evidence that 7-day working does not affect weekend mortality, and emphasised the need for the 7-day service policies and their impact on patient outcomes to be tracked.^6^ This highlights the importance of a rigorous evaluation of the impact of 7-day working in specialist palliative care in terms of patient outcomes.

Access to palliative care, and receipt of palliative care interventions, is associated with changes in health care use (such as less use of acute healthcare) and lower costs, but with improved quality of life and lower symptom burden.^7–9^ Hospital costs are lower for patients seen by palliative care teams, and this association is greater for those with a primary diagnosis of cancer and for those with more comorbidities.^10^ Economic analyses of hospital-based palliative care consistently report a cost saving effect when compared with usual care, but it is less clear how these savings are achieved. For example palliative care patients and matched comparators spend the same amount of time in hospital, but the intensity of care appears reduced.^11^ However, there is a paucity of economic evaluations and cost analyses to enable an understanding of the costs of palliative care, and to guide decision making about different types of services.^12^

There is an international need to understand the impact of different ways of providing care on the key outcomes and health care usage for people at palliative and end-of-life stages. Not only should care be of high quality and cost effective, but it should also meet the needs of those requiring care. The purpose of this study was to evaluate the effect of enhanced 7-day specialist palliative care services on healthcare utilisation, and patient, family carer and staff experiences of care provided.

## Methods

### Aim

To evaluate the effectiveness and effect of enhanced 7-day specialist palliative care services, and to explore a range of perspectives on the enhanced services.

### Design

An exploratory longitudinal mixed-methods convergent research design. The two elements were first a quasi-experimental uncontrolled pre-post quantitative study using patient-level pseudonymised routine data. Second, post-intervention semi-structured interviews to integrate service use data with the perspectives of care users and providers.

### Setting

The two UK localities involved in this work were geographically large (250–350,000 population) and diverse (82% and 95% white respectively, both being in the 20% of most deprived local authority areas). Specialist palliative care services were funded to provide enhanced 7-day services across their healthcare geography, including acute hospital care and community-based services (referred to as sites). The planned enhancements included increasing the numbers of staff working at the weekends and out-of-hours, and changes in skill-mix.

#### Enhanced 7-day service models

The services studied had a degree of flexibility about how they developed their services as part of this project across both acute and community sectors. All were already providing a 7-day service, the aim of the service developments was to enhance these services, through increasing staff numbers and developing new models of working. In location 1 they adopted a nurse-led staffing model, introducing a senior nurse role to focus on complex patients and supporting their specialist palliative care team (a team of Band 8a, 7 and 6 nurses within each of three geographical clusters). Medical support was available, with consultants working one weekend in four. In location 2 they adopted a physician-led staffing model, with additional medical staff employed (two locum staff), supported by more junior nursing staff (two band 7 nurses). Staff recruitment was a particular issue for this team, hence the new nursing staff worked specifically in an urgent response mode.

### Population

The populations of interest in this study included patients receiving specialist palliative care, their family carers and the specialist palliative care staff working within the localities.

For the quantitative data the aim was to include a full-population study of all those who had received care within the study period. For the qualitative data the inclusion and exclusion criteria are described in Table 1.

**Table 1.** Study inclusion and exclusion criteria for qualitative data.

Inclusion criteria	Exclusion criteria
Patient criteria	
Aged ⩾18 and over	Aged <18
Individuals referred to specialist palliative care services at one of the two localities	Lacks capacity to agree to participate in the research, as assessed by clinicians providing the service
	Unable to participate in a qualitative interview using English, as assessed by clinicians providing the service
Family carer criteria	
Identified (by the patient) as a family/informal carer of the person accessing specialist palliative care services at one of the two localities. A broad definition of ‘family’ was used, including those related through committed heterosexual or same sex partnerships, birth or adoption and others who have strong emotional and social bonds with the patient. Carers, who may or may not be family members, were defined as lay, unpaid people in a close supportive role who share in the illness experience of the patient.	Paid carers
Aged ⩾18 years, no maximum age	Lacked capacity to agree to participate in the research, as assessed by staff providing the service or the person taking consent
	Unable to participate in a qualitative interview using English, as assessed by clinicians providing the service
Specialist palliative care staff criteria	
Involved in the provision of specialist palliative care within the two localities, including senior nurse practitioners, advanced nurse practitioners, palliative medicine consultants, consultant nurse practitioners.	
Aged ⩾18 years, no maximum age	

### Sample and sampling

The quantitative component adopted total population sampling whereby data from all adult people (18+ years) accessing the specialist palliative care services within the two localities for set periods were collected retrospectively (for baseline data pre-intervention) and prospectively.

In the qualitative component people using the services post-intervention were purposively sampled to maximise variety based on gender, diagnosis, living status, and amount and type of contact with the service. Participating individuals were asked to invite a family carer of their choice to also take part in the study, although not being able to identify a participating family carer did not preclude study involvement, and identified family carers could participate if the person using the service was subsequently unable to take part. Staff involved in the provision of specialist palliative care within the two localities were purposively sampled to maximise variety based on gender, role and setting in which they work.

### Recruitment

The quantitative component used pseudonymised pa-tient-level data covering the whole population of those who had accessed the services being studied. For the qualitative component service providers were provided with participant packs to introduce the study to people who met the inclusion criteria. Once potential patient participants indicated they were happy to learn more about the research, a purposive sample was drawn to ensure variability in age, gender, diagnosis, amount of contact with the 7-day specialist palliative care service and whether the person lived alone. Full written consent was taken prior to participation by the researcher (SV). Patient participants were asked to pass a recruitment pack to a family or informal carer of their choice. Staff providing the 7-day services within the two localities were provided with invitation packs by the research team to enable them to consider participation in interviews.

### Data collection

#### Quantitative data

Consisted of routinely collected data relating to adults accessing specialist palliative care services in the localities studied over a 12-month period. Data were collected within four time periods: T0 (Baseline), and three (4 months) intervention periods (T1, 2, 3). The Health Research Authority precedent set criteria preferred method for access to patient data was followed, whereby the direct care team extracted and pseudonymised the information. Data extracted are summarised in Table 2.

**Table 2.** Quantitative data collection.

Type of data	Source	Timing^a^
Diagnosis	e-System	Baseline
Primary diagnosis		
Co-morbidities		
Date of diagnosis		
Date of referral to specialist palliative care service	e-System	Ongoing
GP practice	e-System	Baseline/ongoing
Functional status	AKPS	Baseline/ongoing
Ethnicity	e-System	Baseline
Year of birth	e-System	Baseline
Social conditions (partial post-code)	e-System	Baseline
Usual place of residence	e-System	Baseline/ongoing
Gender^b^	e-System	Baseline
Social support (spouse, living alone, not living alone, other family member, residential care home, nursing home)	e-System	Baseline
Place of death (POD)	e-System	Ongoing
Place of care (POC)	e-System	Ongoing
Achieved preferred place of death (PPD)	e-System	Ongoing
Achieved preferred place of care (PPC)	e-System	Ongoing
Number of emergency hospital admissions	e-System	Ongoing
Avoidable admission to hospital (planned/unplanned, length of admission, from emergency department)	e-System	Ongoing
Previous admissions to hospital	e-System	Retrospective
Health status	AKPS	2 weekly (daily with deterioration)
Phase of illness	OACC	2 weekly (daily with deterioration)
Symptoms and concerns	IPOS	2 weekly (daily with deterioration)
Face-to-face review by service clinician	e-System	Ongoing
Calls to weekend/after 5 pm advice line	e-System	Ongoing
Level of intervention (face-to-face assessment, prescription, symptom control, internal referral, external referral, psychological support, rapid discharge, phone call to coordinate care, discharge to hospital/hospice)	e-System	Ongoing
Referral response time	e-System	Ongoing
GP out of hours	Site	Ongoing
Hospice care	Site	Ongoing
Number of days as inpatient		
Day care visits		
Outpatient appointment		

AKPS: Australia-modified Karnofsky Performance Status^13^; OACC: Outcome Assessment and Complexity Collaborative^14^; IPOS: Integrated Palliative Outcome Scale.^15^

a Note, this table indicates the agreed timings, however in practice this varied across sites and across the study.

b The term gender is used here as this was the label used in the pseudonymised data received.

The data were collected from Site Hospital 1 and 2 and Site Hospice 1 and 2, between October 2018 and September 2019.

#### Qualitative data

*Patients and family carers:* Single, conversational, semi-structured interviews were conducted with patient participants at a place of their choosing by SV. An evolving, iteratively developed, topic guide enabled interviews to be primarily driven by participant issues. Where possible, separate interviews with carers were held on the same occasion as the interview with patient participants, but interviews could also be held jointly if preferred. Interviews with patients and carers explored their experiences of the enhanced 7-day service, what was helpful, and what could be improved.

*Service staff:* Individual face-to-face interviews were conducted with staff involved in the provision of specialist palliative care within the two localities at two time points by SV. Topics included exploring their experiences of providing the 7-day services, what they have learnt from the experience, and their perceptions of the impact of the enhanced service.

### Data analysis

#### Quantitative data

The population were described in terms of age, gender, social conditions, social support, religion and ethnicity and characterised in terms of the most frequent primary diagnosis, functional status, health status and symptoms and concerns. Information regarding missing data of each variable can be found in Supplemental Materials. A descriptive statistical analysis was performed allowing for comparison before and after the enhanced 7-day service. A complementary regression analysis was used to understand what contributes to the hospital length of admissions, number of admissions, number of remote contacts with healthcare services, changes in health status and place of death.

An econometric model accounting for possible confounding factors that may be driving variations was developed. The model follows the simple equation: $Y_{ist}=Treatment_{t}+\;X_{ist}+Site_{s}+Period_{t}+u_{ist}$ . Where $Y_{ist}$ is the outcome variable of individual i at site s and period *t*. $Treatment_{t}$ is the treatment binary variable, taking the value 1 if the period of observation falls in the treatment period and 0 otherwise. $X_{ist}$ corresponds to the list of covariates which includes age, age squared, gender, gender and age interaction, a binary variable for race (1 if white British/Irish, 0 otherwise), a binary variable for cancer (1 if cancer, 0 otherwise) and a binary variable for death (1 if dead, 0 otherwise). $Site_{s}$ indicates the site where the individual was observed. $Period_{t}$ contains the possible periods of 4 months and the year. $u_{ist}$ is the normally distributed error ( ~N(0,σ) ) term accounting for the unobservable component of the model. The main variable of interest is $Treatment_{t}$ , which indicates the expected effect of the intervention (i.e. the enhanced 7-day services). $X_{ist},\;Site_{s}\;and\;Period_{t}$ aim at accounting for possible confounders that may arise from heterogeneity in demographics and severity from control to treatment period, seasonality and site scale. Note that by including the year and the four-month periods fixed effects, the model compared the pre-intervention period with the equivalent post intervention period.

#### Qualitative data

The qualitative data were analysed thematically with the aid of NVivo™ qualitative software. Identification of themes and initial coding was undertaken by three members of the evaluation team (SD, ZC and SV), and shared and agreed with SB and CW during evaluation team data analysis meetings. This allowed for the ongoing review, modification and verification of the coding framework and enabled the evaluation team to develop an analysis of the situation.^16^

### Ethics

NHS REC approvals were granted (REC reference 18/NW/0852, 25.1.19), together with research governance approval from all participating organisations. Attention was paid to pseud-anonymisation of routine data, and a clear distress protocol followed when interviewing patients and family carers.

## Results

While in reality the intervention started earlier in some sites and was slowly scaled up in others, for the purposes of this analysis, we used an intervention start date of 1st October 2018. T0 (Baseline) included data between June 2018 to September 2018. The intervention period included T1 (October 2018 to January 2019); T2 (February 2019 to May 2019) and T3 (June 2019 to September 2019).

Qualitative interviews were conducted in 2019 to 2020. A total of 95 interviews were conducted. Nineteen interviews took place with patients, and 23 interviews with family carers. Thirty-three initial interviews were undertaken with staff members across the four sites at time-point 1, with 20 follow-up interviews completed at time-point 2. All data were collected prior to the impact of COVID-19 on care provision.

### Quantitative data

**Figure 1.**
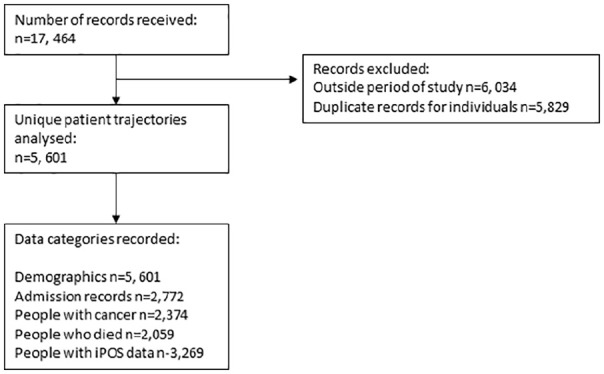
STROBE diagram of quantitative data collection.

Data are presented here on the characteristics of those receiving care. The study includes a total of 5601 unique individuals, from which 1507 belong to the group observed before the new services commenced (June 2018 to September 2018) and 4094 belong to the group observed after the new services commenced (October 2018 to September 2019). Details are presented in Figure 1.

#### Baseline and descriptive data

Generally, people receiving care were in their early old age (mean age 73 years), balanced in gender (49% female vs 51% male), predominantly White British/Irish (90%) and with a high incidence of cancer (42%). The percentage of those who died within the observed period was 37% (Table 3). The distribution per periods of four months is similar, with the lowest share being 21% while the largest being 28%. The locality distribution was slightly less balanced. There is little apparent change in the overall characteristics of those accessing the services before or after the intervention.

**Table 3.** Overall characteristics of those observed in the study.

	Total		Before		After	
	*N*		*N*		*N*	
Total	5601	100%	1507	100%	4094	100%
Age (*N*, average)	5500	73.54	1,507	72.45	3993	73.95
Missing age data	101	2%	0	0%	101	2%
Female	2754	49%	740	49%	2014	49%
Male	2847	51%	767	51%	2080	51%
White British/Irish	5021	90%	1346	89%	3675	90%
Other	324	6%	117	8%	207	5%
Missing ethnicity data	256	5%	44	3%	212	5%
Has cancer	2374	42%	611	41%	1763	43%
Does not have cancer	1472	26%	201	13%	1271	31%
Missing diagnosis data	1755	31%	695	46%	1060	26%
Died during data collection period	2059	37%	612	41%	1447	35%
Did not die during data collection period	3542	63%	895	59%	2647	65%
Locality 1 Community	1303	23%	419	28%	884	22%
Locality 1 Hospital	1936	35%	460	31%	1476	36%
Locality 2 Community	937	17%	324	21%	613	15%
Locality 2 Hospital	1425	25%	304	20%	1121	27%
June 2018 to September 2018	1507	27%				
October 2018 to January 2019	1562	28%				
February 2019 to May 2019	1330	24%				
June 2019 to September 2019	1202	21%				

This table provides the main characteristics of the individual observed in the dataset. The data was collected from Site Hospital 1 and 2 and Site Hospice 1 and 2, between October 2018 and September 2019.

Table 4 displays the change in outcomes from before and after the new service models were instituted.

**Table 4.** Outcomes measured during the study before and after the new service models.

Outcomes	Before			After			Variation	
	*n*	Average or %	SD	*n*	Average or %	SD		
Average length of stay	728	17.22	25.84	1982	16.23	24.39	−0.99	Down
Admissions	730	3.38	10.24	2042	4.87	16.88	1.49	Up
Face-to-face	941	5.71	6.07	3422	4.81	6.59	−0.91	Down
Weekend face-to-face	270	2.52	2.07	983	2.42	2.19	−0.1	Down
Remote interactions	205	13.57	29.31	779	7.01	18.43	−6.56	Down
Weekend remote interactions	21	3.86	3.02	81	3.74	3.55	−0.12	Down
Number of diagnoses	810	3.06	3.53	3006	4.56	4.43	1.5	Up
Phase changes	478	1.23	1.23	1996	1.04	1.15	−0.19	Down
AKPS	709	25.30%	29.67 p.p.	3260	9.92%	21.22 p.p.	−15.38 p.p.	Down
Pain	660	1.61	1.23	2609	1.42	1.20	−0.19	Down
Shortness of breath (SOB)	662	2.26	1.31	2610	2.19	1.25	−0.07	Down
Weakness	632	2.2	1.15	2492	1.92	1.17	−0.27	Down
Depressed	464	1.08	1.34	1685	1.21	1.40	0.13	Up
Peaceful	278	2.24	1.29	925	2.33	1.26	0.09	Up
Preferred place of death (PPD) achieved	252	59%	49 p.p.	1030	70%	46 p.p.	0.11 p.p.	Up
Died in hospital	463	62%	49 p.p.	1259	49%	50 p.p.	−0.12 p.p.	Down

This table reports the number of observations, averages/percentages and standard deviations of the outcome variables analysed in this study in the periods before and after the intervention being implemented. The data was collected from Site Hospital 1 and 2 between October 2018 and September 2019. Note 1: The variables ‘PPD achieved’ and ‘Died in hospital’ are binary variables, which means that the number of observations contains both people that achieved PPD and the ones that did not, as well as the ones that died in hospital and the ones who did not. The column with the percentages tells how many people from the total observed effectively achieved PPD or died in hospital. Note 2: All the remaining variables display simple averages. Note 3: Pain, SOB, Weakness, Depressed and Peaceful are categorical variables whose values range from 0 to 4, where 0 means ‘no problems’ and 4 means ‘overwhelming problems’.

Average length of stay decreased by 1 day in the time after the new service models started, while the average number of admissions per patient increased by 1.5 admissions. The number of other interactions, such as face-to-face consultations decreased, both generally and at the weekend. The Australian Modified Karnofsky Performance Status (AKPS) score presented a significant decrease, of more than 15 percentage points, indicating that patients seen during the intervention period had a poorer performance status. However, the self-reported results in the iPOS dataset are mixed. Pain, shortness of breath and weakness are reported at a lower average, while depressive and peace states are reported at a higher average. More people died in their preferred place of death and fewer in the hospital. Note, however, that these are only overall variations, and may or not be because of the commencement of new service provision.

Table 5 displays four econometric models, estimated by Ordinary Least Squares, examining outcomes for average length of hospital stay and number of hospital admissions, as an example. The first column shows a model which includes only demographic covariates. The second column adds site fixed effects to the model when we add Site 2 Hospital as a variable of interest in the regression. The third column adds period fixed effects to the model in column 2. The fourth column adds severity variables in the form of cancer and death. Each observation refers to a patient. The treatment variable, in all four variations of the model, shows a negative but non-statistically significant variation. According to model 4, the most detailed explanatory model, on average, patients in the treatment period stay in hospital 0.16 days less but are hospitalised 2.67 more times. Each additional year of age increases length of stay by 0.6 days and number of admissions by 0.28 times. Females stay almost 3.5 more days in the hospital, but are admitted 2.48 less times, on average. People with cancer have shorter hospitalisations, with 4 days fewer than other patients, and have approximately two fewer admission episodes than other patients, on average. A summary of all regressions for 16 outcomes is presented in table 6.

**Table 5.** Changes in hospital length of stay, and number of hospital admissions during the intervention study period.

	Length of stay				Number of hospital admissions			
	Model 1	Model 2	Model 3	Model 4	Model 1	Model 2	Model 3	Model 4
Post-intervention period	−0.765 [−2.831;1.300]	−1.425 [−3.517;0.668]	−0.265 [−4.941;4.411]	−0.161 [−4.669;4.347]	1.666 [0.615;2.718]	1.114 [0.091;2.137]	1.367 [−1.536;4.270]	2.669 [−1.094;6.432]
Age	−0.34 [−1.087;0.407]	−0.286 [−1.026;0.453]	−0.282 [−1.026;0.463]	0.61 [0.069;1.151]	0.232 [−0.045;0.509]	0.271 [−0.004;0.547]	0.282 [0.008;0.557]	0.277 [−0.089;0.643]
Female and Age	−0.104 [−0.356;0.147]	−0.104 [−0.354;0.146]	−0.104 [−0.355;0.147]	−0.0596 [−0.221;0.103]	0.021 [−0.070;0.112]	0.022 [−0.067;0.112]	0.023 [−0.067;0.112]	0.0262 [−0.083;0.135]
Age squared	0.002 [−0.003;0.008]	0.002 [−0.003;0.007]	0.002 [−0.003;0.007]	−0.00442 [−0.008; −0.001]	−0.002 [−0.004; −0.000]	−0.003 [−0.005; −0.001]	−0.003 [−0.005; −0.001]	−0.00285 [−0.005; −0.0004]
Female	7.794 [−12.078;27.665]	7.77 [−12.008;27.548]	7.809 [−12.056;27.675]	3.422 [−9.232;16.077]	−2.208 [−9.613;5.198]	−2.297 [−9.636;5.043]	−2.316 [−9.649;5.017]	−2.476 [−11.463;6.511]
White British/Irish	−0.208 [−5.787;5.371]	0.517 [−5.037;6.071]	0.492 [−5.090;6.073]	−3.708 [−13.025;5.609]	−3.88 [−9.492;1.731]	−3.318 [−8.907;2.271]	−3.394 [−8.987;2.199]	0.0543 [−5.267;5.376]
Site 2 Hospital		−5.748 [−7.315; −4.181]	−5.719 [−7.365; −4.072]	−6.094 [−8.033; −4.155]		−4.55 [−5.324; −3.775]	−4.834 [−5.769; −3.900]	−6.094 [−8.033; −4.155]
October to January			−0.684 [−4.841;3.473]	−0.451 [−4.294;3.392]			0.633 [−1.895;3.162]	−0.98 [−4.349;2.389]
February to May			−0.885 [−4.567;2.798]	0.14 [−3.29;3.57]			0.872 [−1.143;2.888]	−1.284 [−3.99;1.422]
2019			−0.825 [−3.598;1.948]	−1.382 [−4.532;1.768]			−1.233 [−3.656;1.191]	−1.675 [−4.344;0.994]
Has cancer				−4.312 [−6.615; −2.009]				−1.932 [−3.744; −0.12]
Has died				0.742 [−1.951;3.435]				−0.173 [−2.167;1.822]
Number of observations	2632	2632	2632	1561	2694	2694	2694	1595

This table displays the coefficients and confidence intervals of the Ordinary Least Squares regressions for the effect of the intervention on Length of Stay and Hospital Admissions. The standard errors are robust and clustered at the individual level. The regressions use data from Site Hospital 1 and 2, collected between October 2018 and September 2019. Column 1 should only be interpreted as the right model if it is believed that site characteristics, seasonality and severity do not play a role in explaining changes in length of stay from control to treatment. Column 2 should only be interpreted as the right model if it is believed that seasonality and severity do not play a role in explaining changes in length of stay from control to treatment. Column 3 should only be interpreted as the right model if it is believed that severity does not play a role in explaining changes in length of stay from control to treatment.

**Table 6.** Meta table showing effects of the new model of service provision on selected outcomes.

Outcomes	Model 1		Model 2		Model 3		Model 4	
	Signal	Significance	Signal	Significance	Signal	Significance	Signal	Significance
Average length of stay	Down	No	Down	No	Down	No	Down	No
Admissions	Up	Yes	Up	Yes	Up	No	Up	No
Face-to-face	Down	Yes	Up	No	Up	No	Down	No
Weekend face-to-face	Down	No	Up	No	Down	No	Down	No
Remote interactions	Down	Yes	Down	Yes	Down	No	Up	No
Weekend remote interactions	Down	No	Up	No	Down	No	Down	No
Number of diagnoses	Up	Yes	Up	Yes	Up	Yes	Up	Yes
Phase changes	Up	No	Up	No	Up	No	Up	No
AKPS	Down	Yes	Down	Yes	Down	Yes	Down	Yes
Pain	Down	Yes	Down	No	Down	No	Down	No
SOB	Up	Yes	Up	No	Down	Yes	Down	Yes
Weakness	Down	Yes	Down	No	Down	No	Down	No
Depressed	Up	No	Up	No	Up	No	Up	No
Peaceful	Up	Yes	Up	No	Down	No	Down	No
PPD achieved	Up	No	Up	No	Up	No	Up	No
Died in hospital	Down	Yes	Down	Yes	Down	No	Down	No

This meta table summarises the results of the Ordinary Least Squares (Length of Stay, Admissions, Face-to-face, Remote interactions, Weekend remote interactions, Number of diagnosis, Phase changes and AKPS), Ordered Probit (Pain, SOB, Weakness, Depressed and Peaceful) and Probit (PPD Achieved and Died in hospital) regressions for the effect of the intervention on all the outcomes in analysis. All regressions have robust standard errors, clustered at the individual level. The regressions use data from Site Hospital 1 and 2, collected between October 2018 and September 2019. Model 1 includes only demographics. Model 2 adds site fixed effects to model 1. Model 3 adds year and four months fixed effects to model 2. Model 4 adds cancer and dead binary variables to model 3. Statistical significance is defined with 95% confidence level.

### Qualitative data

The characteristics of those participating are displayed in Tables 7 and 8. Five interrelated themes were identified through the analysis of interviews with patients, family carers and staff: Responsiveness (of the service); Reassurance (patient, family carers and staff); Relationships (between patient, family carers and staff; within staff groups and with other stakeholder groups); Reciprocity (between patient, family carers and staff; within staff groups and with other stakeholder groups) and Retention (of service staff).

**Table 7.** Demographic characteristics of patient and family carer participants.

Characteristic	Patient participants *n* = 19	Family carer participants *n* = 23
Gender	M = 11, F = 8	M = 7, F = 16
Age (in years)	Mean age 67 (range 39–91)	Age range 21–89
Lives alone	*n* = 8	–
Diagnosis	Cancer *n* = 14	–
Relationship with patient	–	Partner (incl. spouse, ex-spouse) = 15
		Child = 7
		Other relation = 1
Locality	Locality 1 *n* = 8	Locality 1 *n* = 9
	Locality 2 *n* = 11	Locality 2 *n* = 14

**Table 8.** Demographic characteristics of staff participants.

Staff participants	*n* = 33
Gender	M = 3, F = 33
Professional role	Consultant in palliative care *n* = 3
	Palliative care support worker *n* = 1
	Service manager *n* = 2
	Specialist palliative care doctor *n* = 2
	Specialist palliative care nurse *n* = 24
	Specialist palliative care administrator *n* = 1
Years working in palliative care	Mean 10.5 (range 1–24)
Years in current post	Mean 3.2 (range 0.08–18)
Locality	Locality 1 community *n* = 9
	Locality 1 hospital *n* = 9
	Locality 2 community *n* = 8
	Locality 2 hospital *n* = 8

#### Responsiveness

‘Responsiveness’ was evident in two main outcomes-related themes: the ability to identify and respond to individual patient needs; and the ability to respond in a timely manner to these changing needs. The increased responsiveness appeared to be a direct result of the additional capacity afforded by the enhanced 7-day programme:

[There is now] overall a quicker response and a quicker access to us [. . .] The team did see patients pretty quickly anyway but due to the increased levels of staffing, patients can be seen a lot more quickly and a lot more support can be given to relatives or even the patient, just ‘cos there’s more time to be able to do that. (Staff member 24, Int2)

The increased capacity within the specialist palliative care teams had provided staff with more time, enabling them to identify and address patient and carer needs. This was important to patients and their families:

In fact it was [the specialist palliative care nurse] that spotted that I was ill [. . .] She came one day, I didn’t ask her to, she just came one day, and she looked at me and she said, ‘You’re not looking very well’ she said, ‘we’ll send you in for blood transfusions’. So I had four of them in a week [. . .]. (Patient A)

As a result of increased medical support, a patient’s needs could be met during a weekend rather than having to wait until Monday:

Sometimes it’s just the very complex medical issues, you know really complex pain control, really complex symptoms, but sometimes it’s just that need for a senior medic to be able to make decisions [. . .] and often it would just end up being kind of left ‘til the Monday to the detriment of the patient, but I think with that senior medical presence on a Sunday [it has] made a difference. (Staff member 12, Int2).

As the seven-day programme progressed, the increase in capacity and its impact on service responsiveness became visible across all days of the week, not only on days 6, 7 and public holidays. With more staff members, for example, acute and community teams were able to change working hours and provide the specialist palliative care service for longer hours on weekdays:

I think it’s really turned around the kind of quality of care that we can offer in [the] community. The extension of the hours has been something that’s been really good, having the eight ‘til six service, because patients do invariably ring at five o’clock on an evening to say that somebody’s had a bad day and you know you need this, I need it sorting out, you’ve still got that time to do that. (Staff member 17, Int2)

#### Reassurance

Both patients and family carers were reassured by the availability of the service. Some participants were unable to recall whether they had used the specialist palliative care service out-of-hours; when they felt unwell, they could ‘forget the day and time’, and the details of the service they had used became irrelevant to them. Whatever the day, it was important to patients and family carers that the specialist palliative care service was available should they need it:

We’ve phoned on a Sunday and [the specialist palliative care team] has come out [. . .] You were really struggling weren’t you with your breathing and pain [. . .] It gives you peace of mind. That you know there’s somebody knowledgeable at the end of the phone. (Patient K and Family Carer P)

Patients, family carers and staff felt reassured as a result of the responsiveness of the specialist palliative care teams. During the illness experience, patients and family carers experienced uncertainty and fear, making the reassurance provided by the specialist palliative care service all the more comforting:

I don’t have that fear of pain anymore because of them [. . .] . . . I’ll be honest, it’s more than important. I would say it’s taken about 80% of my worries and fears away. (Patient G)

For staff, increased reassurance was the result of a number of changes brought about by the 7-day programme. A major source of reassurance came from working alongside other colleagues at weekends, rather than alone – both for the increased capacity and the peer support this provides:

I remember the A&E [emergency department] nurse – I think her comment was ‘Thank goodness you’re here’, and it was very much like relief of ‘I didn’t know what to do, I was out of my depth’. (Staff member 12, Int2)

#### Relationships

The relationships that patients and family carers had with members of the specialist palliative care teams were meaningful to them, particularly continuity of care from the same specialist palliative care team members. All participants referred to their ‘key contact’ within their respective specialist palliative care service, with this positive experience of continuity of care was often contrasted with their experiences of other services. They felt understood and did not have to re-tell their history and symptoms, or repeat themselves ‘to different people’:

I would be completely lost without it [Enhanced service] to be honest with you, feel lost. I’d be panicking. GPs are ok but you sort of see a different one every time that they come. (Family Carer L)

The relationships and trust that patients had with their specialist palliative care nurse meant they had ‘somebody to turn to’ outside their immediate family, so they could protect their loved ones. Staff noted that the increased capacity of specialist palliative care and the integration of hospital and community services allowed them to develop more supportive relationships with patients and family carers:

Symptomatically she was fine, she didn’t have any pain, she didn’t have any sickness or any other traditional kind of palliative symptoms at all, but what she did have was worry about kind of advance care planning type issues, so I think maybe before she wouldn’t necessarily have been prioritised on any day, . . . just never reach the top of that list if that makes sense. . . . but I was able to go to her almost every day . . . and have both conversations about what she was worried about and what she was worried about for her family and you know that kind of it shouldn’t be extra but when a team’s pressurised becomes extra doesn’t it. (Staff member 4, Int2)

The 7-day programme also had a positive impact on relationships between specialist palliative care staff and other healthcare professionals. Increased capacity enabled acute and community specialist palliative care teams to gain greater visibility and increased profiles in healthcare teams, often because ‘people have heard about the [specialist palliative care] service more’:

You sort of raise your profile when you’re there every day, . . .. before they might have just given us everybody and not taken any ownership and but now they’re saying actually you don’t need to do that one, so I think the teams are better and they’re giving us definitely better referrals. (Staff member 19, Int2)

Before we did seven day working, nobody sent a referral at the weekend, and so everybody said well why do we need to do seven-day working because we don’t get any referrals at the weekend? . . .people will only demand things when it might be deliverable. . .So there’s something about the visibility and the referrals. (Staff member 32, Int1)

#### Reciprocity

Patients and family carers explained how specialist palliative care staff took time to listen to them, valuing their knowledge and expertise. This resulted in joint decision-making between patients, carers and staff – where knowledge and expertise were reciprocal:

Well first of all they listen, and then they start suggesting solutions, they only suggest them, they don’t say we’re doing this or we’re doing that, they suggest solutions [. . .] And they listen to what you’re saying, like you’re listening to me now. So it’s not as if they listen and go, you know, they’re actually listening, you can always tell when somebody’s actually listening to what you’re saying. (Patient N and Family Carer Y)

In other examples, specialist palliative care staff worked closely with other healthcare teams to meet patients’ needs, such as managing symptoms and achieving preferred place of care:

Family Carer: It’s [generalist staff] that cause the real problem to be honest. It was like getting discharged [from hospital] or, you know, the fight between [the specialist palliative care team] and the hospital doctors when we were trying to go home, and it was a case of them saying ‘Oh, we wanted to keep you in for another day to observe you’, and [the specialist palliative care team] were like, ‘Well, no this is just about the cancer, she might as well go home’. . . .. . .. (Family Carer S)

#### Retention

The creation of additional skill mix, particularly of more senior nursing roles, was an important factor in retaining staff within the services. In addition to enhancing patient care, nursing staff appreciated the opportunity that the senior role provided for career progression, giving them an option to remain in a clinical role while gaining promotion:

So I think having the grading between an eight, a seven and a six, I think that is important . . . because then you’ve got scope for career progression. [In the past when working in a hospice] you were a staff nurse or a sister, you had [no progression opportunities], and sisters stayed there for years, they never went anywhere. (Staff member 1, Int2)

In addition, having a mix of skills within the teams, and the ‘*right*’ people to deliver the specialist palliative care service improved team working and well-being:

We’ve got some brand-new staff members with their new ideas and the enthusiasm and different ways of looking and approaching care and different ideas, which is you know motivating and, you know, increases morale. It feels like they enjoy the job and that . . . they’ve picked the right people for the job you know. (Staff member 27, Int2)

The seven-day programme enabled three of the sites within the localities to proactively develop their specialist palliative care service, whereas previously staff had felt they were ‘*fire-fighting*’ and only able to focus on meeting the demands of their caseloads.

## Discussion

### Summary

The purpose of this evaluation was to examine the effect and implications of investments in specialist palliative care to provide an enhanced 7-day service, where additional staff capacity enabled a more comprehensive and intensive service throughout the week. The qualitative data suggest that enhanced support was well received by staff within and outside the specialist palliative care services, and valued by patients and family carers. Patients and family carers were satisfied with the services which appeared to increase their confidence and help them to be less fearful. They were enabled to be more active partners in decision making. The enhancements also had positive effects on staff: increasing their confidence, knowledge and skills, and enabling job satisfaction and team working. The skill mix brought about by the enhanced services were seen as integral to these positive effects, particularly role and importance of senior nurses within the team. Perhaps the most important aspect of the enhancement, for all participants, was that it enabled relationships, and these were highly valued. For patients and carers there was an opportunity to build relationships with staff, enabling continuity of care and deeper levels of collaboration between patients/carers and staff. For specialist palliative care staff, the development of relationships within the services brought assurance and reassurance, whilst out with the services staff felt seen and valued by other health professionals, which in turn raised the profile of the enhanced services and its staff.

### What this study adds

The quantitative data show a trend towards a decreased length of stay in hospital, but with a likelihood of being admitted more frequently. Generally, those using services appeared to have characteristics that would indicate they are more unwell. There appear to be some differences in service use depending on age (each additional year of age increased length of stay by 0.6 days and number of admissions by 0.28 times), gender (women had longer lengths of stay, but fewer admissions than men) and whether people have cancer (resulting in shorter hospitalisations). Studies that investigate the impact of gender on rates of readmission mostly identify that females are more likely to be readmitted within 30 days of discharge than males^17–19^; the data from this evaluation identify gendered patterns of hospital usage, but potentially in different patterns than previously known.

Enhancing the service meant that those already likely to be benefitting from service access appear to continue to do so, rather than necessarily reaching other populations who might benefit from palliative care. For example, the enhanced service does not appear to have made much difference to the proportions of those using the services with different underlying diagnoses (although this is hard to determine due to missing, and complex, diagnosis information), or from minority ethnic populations, although this may have been impacted by the demographics of the localities. It is known that specialist palliative care services can struggle to reach out to underserved populations.^20^ Given the importance of improving access to underserved populations, future service enhancements need to clearly identify who these populations are and design service amendments appropriately, in collaboration with, or led by, the communities that existing services are not serving.

A predominant driver of healthcare cost towards the end of life is inpatient hospital use,^21^ and the likelihood of using hospital services increases the closer someone is to death.^22^ People, however, express a strong preference to stay at home, for as long as possible.^23^ At the same time, there is recognition that hospitalisation is an important component of care. Despite the association between palliative care and lower levels of healthcare utilisation,^7^ the evidence on its effects on length of stay is more equivocal. An early review intimated palliative care had no effect on length of stay in hospital^24^ however subsequent work has shown a reduction in length of stay in intensive care,^25^ or more generally.^26^

There is evidence that palliative care is associated with less costly hospitalisations perhaps because of reduced lengths of stay, reduced intensity of treatment during stay or a combination of both.^24,27^ The cost saving-effect of palliative care teams also has been found to be larger for people with cancer with high numbers of multi-morbidities, such as in this population.^28^ In this evaluation, a trend towards a reduced length of stay when in hospital is noted. This is likely to be positively evaluated by patients, and will also reduce hospitalisation costs, however data on intensity of treatment during hospital stay are important in unpicking important components associated with the model and associated costs or cost savings. Further research measuring intensity of treatment and skill mix of the teams delivering care would contribute to shed light on this topic.

The qualitative data from this study indicate an impact on patients who valued the responsiveness, reassurance, relationships and the importance of reciprocity that the enhanced service provided. We know that patients’ experience of care is highly influenced by skills such as communication, involvement in decision making, relationship building and trust,^29,30^ and that these transfer to the bereavement and grief experience of family carers. ‘Slow’, managed care can (re)distribute power and knowledge in important ways related to care experience and quality.^31^ Relationships built over time enable the careful negotiation of preferences and priorities in a way that transcends simple metrics such as ‘place of death’.^32^ The importance here of a mixed methods evaluation cannot be understated. Whilst the objectively measurable impacts of these enhanced services may seem modest, the importance of qualitative work subtly unpicking the way that care is experienced, at such an important juncture in life is vital. The key question, perhaps, is how much we value these aspects of care quality in the current pressured, typically understaffed, care environment.

Senior nursing roles appeared to be important, contributing skills in clinical assessment and prescribing, but also having an impact on recruitment and retention. The support of others in the team, including doctors, was also important. The importance of qualified and senior nurses within nursing skill mix is known; a nursing skill mix with a higher proportion of qualified nursing staff has been shown to improve patient outcomes,^33^ although there is equivocal evidence on the impact of advanced nursing roles.^34^ Nurses can provide equal or better care to doctors in some circumstances, with positive outcomes and high satisfaction.^35^ It is critical therefore to consider by whom, and how, decisions are made about the focus of service enhancements, and if they privilege potentially particular staff.

#### Strengths and limitations

A ‘real world’ uncontrolled before and after (pre-post) design was adopted which is vulnerable to picking up effects of pressures on the system that are unrelated to the implementation of the service.^36^ There could also be ‘intervention creep and spread’, with a much less distinct ‘before’ and ‘after’, for example due to delays in implementing the services as planned because of delays in appointing and training staff. Sustaining the intervention is also challenging, which may mean that it is more challenging to detect an impact where there is one.

The use of routine data can also be challenging, as this can be differentially collected, patchy in places, or absent. This was certainly the case in this evaluation, such as differing interpretations about how data on care outcomes (e.g. iPOS and Phase data) were collected, and also high amounts of missing data both in fields where it might be expected (e.g. recording preferred place of care), but also where it might not have been (e.g. diagnosis). In the qualitative phase we did not sample participants based on ethnicity, and there may be differences in experience that we were unable to explore.

A strength of this mixed-methods approach is the inclusion of narrative accounts collected from those providing and using the services. Whilst we actively interrogated interview data for negative or equivocal experiences, accounts of the experience of the enhanced service model were, for the large part overwhelmingly positive. Patients’ and family carers’ sense of trust in staff, and the confidence that their care would be well-managed by specialist palliative care services is palpable and important. These are not areas that are easy to measure in larger population-based measures, but which are fundamentally important to patients and carers, as well as a source of job satisfaction for staff.

## Conclusions

Seven-day services should continue to be developed or enhanced to provide high quality and integrated palliative care to patients and families. The development and retention of staff should be a key consideration for sites developing similar enhanced services, with attention given to the skill mix, including senior nursing roles. These roles provide expertise to the teams, may be cost-effective, and enable efficient care but are also important in the sustainability of a service by providing career progression whilst remaining in a clinical role. The enhanced services evaluated appear to have an effect on important outcomes such as length of stay and frequency of admission to hospital, but primarily for those who are already typical users of the services. If enhanced services are intended to improve access by under-served populations then it is important that this is clearly articulated and the enhancements carefully designed to facilitate this.

## Supplemental Material

sj-pdf-1-pmj-10.1177_02692163231201486 – Supplemental material for ‘Thank goodness you’re here’. Exploring the impact on patients, family carers and staff of enhanced 7-day specialist palliative care services: A mixed methods study Click here for additional data file. Supplemental material, sj-pdf-1-pmj-10.1177_02692163231201486 for ‘Thank goodness you’re here’. Exploring the impact on patients, family carers and staff of enhanced 7-day specialist palliative care services: A mixed methods study by Catherine Walshe, Céu Mateus, Sandra Varey, Steven Dodd, Zoe Cockshott, Luís Filipe and Sarah G Brearley in Palliative Medicine
