# Supplementary material for: ‘Thank goodness you’re here’. Exploring the impact on patients, family carers and staff of enhanced 7-day specialist palliative care services: A mixed methods study
Source: Palliat Med. 2023 Sep 21;37(10):1484–97. doi: 10.1177/02692163231201486 (PMC10657500; doi:10.1177/02692163231201486)
Supplement: sj-pdf-1-pmj-10.1177_02692163231201486 – Supplemental material for ‘Thank goodness you’re here’. Exploring the impact on patients, family carers and staff of enhanced 7-day specialist palliative care services: A mixed methods study [file sj-pdf-1-pmj-10.1177_02692163231201486.pdf]

Supplementary file.

Site 1 data

Site 1 Community - Overall		
Variables	Number of observations	Average/Percentages
Automated data	1303	100%
Age	1303	71.44
Missing	0	0
Gender		
Female	634	49%
Male	669	51%
Missing	0	0%
Ethnicity		
White British	834	79%
White Irish	27	3%
White Scottish	4	0%
White	19	2%
Mixed British	143	14%
Black British	1	0%
Other European	6	1%
African	4	0%
Indian	5	0%
Asian	5	0%
Other Ethnic Groups	1	0%
Pakistani	0	0%
Missing	252	19%
Religion		
No Religion	41	11%
Not known	3	1%
Not recorded	177	48%
Atheist	4	1%
Protestant	2	1%
Methodist	2	1%
Christian	4	1%
Catholic	37	10%
Church of England	87	24%
Orthodox	0	0%
Congregationalist	1	0%
Jehovah's Witness	2	1%
Jewish	5	1%
Muslim	1	0%
Sikh	1	0%
Missing	936	72%
Interactions		
Face to face consultations	979	5.38
Telephone contact by patient	245	2.45
Telephone contact by relative	558	2.96
Telephone contact to patient	908	6.18
Telephone contact to relative	796	5.69

Site 1 Community - Overall		
Variables	Number of observations	Average/Percentages
Telephone consultations	1213	11.34
Weekend face to face consultations	347	1.85
Weekend telephone contact by patient	57	1.75
Weekend telephone contact by relative	175	1.52
Weekend telephone contact to patient	395	1.93
Weekend telephone contact to relative	352	1.89
Weekend telephone consultations	532	2.61
Diagnosis		
Breast Cancer	43	4%
CNS Cancer	18	2%
Colorectal Cancer	79	8%
Headneck Cancer	57	6%
Lung Cancer	227	23%
Prostate Cancer	8	1%
Gastrointestinal Cancer	68	7%
Urological Cancer	60	6%
Gynaeological Cancer	50	5%
Liver Cancer	16	2%
Skin Cancer	7	1%
Unknown Primary Cancer	19	2%
Pancreatic Cancer	75	8%
Haematological Cancer	0	0%
Renal Cancer	0	0%
Sarcoma Cancer	4	0%
Other Cancer	1	0%
COPD	99	10%
Respiratory	19	2%
Renal	8	1%
CVA	1	0%
Cardiac	0	0%
Heart Failure	33	3%
Dementia	10	1%
MND	6	1%
Parkinson	2	0%
Sclerosis	0	0%
Neurological Other	3	0%
Frailty	1	0%
HIV	0	0%
Haematological Other	31	3%
Liver Failure	6	1%
Other Diagnosis	3	0%
Missing	323	25%
IPOS Data	772	100%
Repetitions in IPOS dataset		
Number of times in dataset	772	3.10
Number of times in dataset on weekends	196	1.19
AKPS	756	53%

Site 1 Community - Overall		
Variables	Number of observations	Average/Percentages
Missing	16	2%
Cancer		
Has cancer	597	77%
Does not have cancer	158	20%
Missing	22	3%
Phase changes		
Phase changes observed	432	1.86
No phase changes observed	368	2.20
Pain		
No pain	144	20%
Slight pain	187	26%
Moderate pain	219	30%
Severe pain	145	20%
Overwhelm pain	37	5%
Missing	39	5%
Weakness		
No weakness	40	6%
Slight weakness	127	18%
Moderate weakness	259	36%
Severe weakness	204	28%
Overwhelm weakness	91	13%
Missing	51	7%
Peace		
No peace	74	11%
Occasional peace	116	17%
Sometimes peace	202	30%
Most times peace	224	33%
Always peace	60	9%
Missing	96	12%
Social support		
External care	2	0%
Homeless	1	0%
Intermediate care	2	0%
Lives alone	186	30%
Lives with family	95	15%
Lives with partner	323	52%
Nursing home	8	1%
Prison	1	0%
Residential	8	1%
Psychiatric hospital	0	0%
Missing	146	19%
Deaths Data	431	100%

Site 1 Community - Overall		
Variables	Number of observations	Average/Percentages
Place of death		
Home	251	58%
Hospice	85	20%
Hospital	76	18%
Nursing	19	4%
Missing	0	0%
Preferred place of death		
Home	269	70%
Hospice	91	24%
Hospital	9	2%
Nursing	12	3%
Missing	51	12%
PPD achieved		
PPD achieved	330	76%
PPD not achieved	51	12%
Missing	52	12%

Site 1 Hospital - Overall		
Variables	Number of observations	Average/Percentages
Automated data	1936	100%
Age	1936	74.23
Missing	0	0
Gender		
Female	957	49%
Male	979	51%
Missing	0	0%
Ethnicity		
White British	1807	93%
White Irish	40	2%
White Scottish	0	0%
White	22	1%
Mixed British	3	0%
Black British	0	0%
Other European	0	0%
African	10	1%
Indian	8	0%
Asian	13	1%
Other Ethnic Groups	8	0%
Pakistani	14	1%
Bangladesh	2	0%
Caribean	4	0%
Chinese	3	0%
Missing	0	0%
Religion		
No Religion	217	11%
Not known	260	13%
Not recorded	0	0%
Atheist	0	0%
Protestant	0	0%
Methodist	40	2%
Christian	30	2%
Catholic	370	19%
Church of England	938	48%
Orthodox	0	0%
Congregationalist	2	0%
Jehovah's Witness	4	0%
Jewish	20	1%
Muslim	31	2%
Sikh	2	0%
Agnostic	3	0%
All Free Church	4	0%
Hindu	5	0%
Mormon	2	0%
Pagan	1	0%
Pentecostal	1	0%
Presbyterian	1	0%

Site 1 Hospital - Overall		
Variables	Number of observations	Average/Percentages
Spirit	1	0%
Salvation Army	1	0%
United Reform Church	3	0%
Missing	0	0%
Interactions		
Weekend face to face consultations	599	3.47
Face to face consultations	1513	8.46
Weekend telephone contacts	202	1.28
Telephone contacts	191	1.17
Remote interviews EPR	749	1.67
Admissions	1926	5.94
Length of stay	1864	18.30
Type of emergency		
Palliative emergency	181	11%
Perceived emergency	70	4%
Normal emergency	449	27%
Missing	931	57%
Diagnosis		
Breast Cancer	22	2%
CNS Cancer	26	2%
Colorectal Cancer	62	6%
Headneck Cancer	43	4%
Lung Cancer	138	12%
Prostate Cancer	13	1%
Gastrointestinal Cancer	75	7%
Urological Cancer	41	4%
Gynaeological Cancer	32	3%
Liver Cancer	8	1%
Skin Cancer	7	1%
Unknown Primary Cancer	28	2%
Pancreatic Cancer	59	5%
Haematological Cancer	0	0%
Renal Cancer	0	0%
Sarcoma Cancer	1	0%
Other Cancer	0	0%
COPD	108	10%
Respiratory	50	4%
Renal	38	3%
CVA	53	5%
Cardiac	0	0%
Heart Failure	59	5%
Dementia	30	3%
MND	2	0%
Parkinson	1	0%
Sclerosis	0	0%
Neurological Other	39	3%
Frailty	19	2%
HIV	0	0%

Site 1 Hospital - Overall		
Variables	Number of observations	Average/Percentages
Haematological Other	44	4%
Liver Failure	17	2%
Other Diagnosis	19	2%
Missing	815	42%
IPOS Data	1637	100%
Repetitions in IPOS dataset		
Number of times in dataset	1637	4.63
Number of times in dataset on weekends	831	2.32
Cancer		
Has cancer	614	38%
Does not have cancer	536	33%
Missing	494	30%
AKPS	1584	34%
Missing	53	3%
Phase changes		
Phase changes observed	1065	131%
No phase changes observed	1108	328%
Pain		
No pain	329	20%
Slight pain	226	14%
Moderate pain	233	14%
Severe pain	140	9%
Overwhelm pain	70	4%
Missing	639	39%
Weakness		
No weakness	149	9%
Slight weakness	183	11%
Moderate weakness	292	18%
Severe weakness	188	11%
Overwhelm weakness	93	6%
Missing	732	45%
Peace		
No peace	157	10%
Occasional peace	119	7%
Sometimes peace	187	11%
Most times peace	146	9%
Always peace	99	6%
Missing	929	57%
Social support		
External care	13	1%
Homeless	0	0%

Site 1 Hospital - Overall		
Variables	Number of observations	Average/Percentages
Intermediate care	0	0%
Lives alone	324	20%
Lives with family	96	6%
Lives with partner	319	19%
Nursing home	54	3%
Prison	2	0%
Residential	33	2%
Psychiatric hospital	1	0%
Missing	795	49%
Deaths Data	842	100%
Place of death		
Home	143	17%
Hospice	48	6%
Hospital	634	75%
Nursing	17	2%
Missing	0	0%
Preferred place of death		
Home	206	35%
Hospice	77	13%
Hospital	263	45%
Nursing	31	5%
Missing	259	31%
PPD achieved		
PPD achieved	434	51%
PPD not achieved	143	17%
Missing	268	32%

Site 2 data

Site 2 Hospital - Overall		
Variables	Number of observations	Average/Percentages
Total	1425	100%
Age	1324	75.45
Missing	101	0.07
Gender		
Female	715	50%
Male	710	50%
Missing	0	0%
Ethnicity		
White British	1382	97%
White Irish	19	1%
White	9	1%
Mixed British	1	0%
Asian British	2	0%
Caribean	1	0%
Indian	1	0%
Other Ethnic Groups	1	0%
Missing	2	0%
Religion		
Missing	1425	100%
Interactions		
Weekend face to face consultations	166	1.17
Face to face consultations	1078	1.40
Length of stay	846	12.52
Admissions	846	1.13
Diagnoses	792	2.44
Diagnosis		
Breast Cancer	49	6%
CNS Cancer	15	2%
Colorectal Cancer	62	8%
Headneck Cancer	44	6%
Lung Cancer	129	16%
Prostate Cancer	9	1%
Gastrointestinal Cancer	43	5%
Urological Cancer	24	3%
Gynaeological Cancer	26	3%
Liver Cancer	19	2%
Skin Cancer	7	1%
Unknown Primary Cancer	13	2%
Pancreatic Cancer	50	6%
Haematological Cancer	5	1%
Renal Cancer	3	0%
Sarcoma Cancer	1	0%

Site 2 Hospital - Overall		
Variables	Number of observations	Average/Percentages
Other Cancer	9	1%
COPD	32	4%
Respiratory	71	9%
Renal	8	1%
CVA	9	1%
Cardiac	0	0%
Heart Failure	31	4%
Dementia	35	4%
MND	2	0%
Parkinson	0	0%
Sclerosis	1	0%
Neurological Other	16	2%
Frailty	7	1%
HIV	1	0%
Haematological Other	32	4%
Liver Failure	12	2%
Other Diagnosis	24	3%
Missing	633	44%
Repetitions in data		
Number of times in dataset	1425	1.41
Number of times in dataset on weekends	262	1.17
Cancer		
Has cancer	617	43%
Does not have cancer	502	35%
Missing	306	21%
AKPS	897	32%
Missing	528	37%
Phase changes		
Phase changes observed	169	122%
No phase changes observed	298	124%
Pain		
No pain	271	19%
Slight pain	165	12%
Moderate pain	174	12%
Severe pain	74	5%
Overwhelm pain	27	2%
Missing	714	50%
Weakness		
No weakness	168	12%
Slight weakness	199	14%
Moderate weakness	183	13%
Severe weakness	90	6%
Overwhelm weakness	23	2%
Missing	762	53%

Site 2 Hospital - Overall		
Variables	Number of observations	Average/Percentages
Peace		
No peace	88	6%
Occasional peace	85	6%
Sometimes peace	123	9%
Most times peace	140	10%
Always peace	38	3%
Missing	951	67%
Social support		
Lives alone with help	338	24%
Lives alone with no help	117	8%
Lives in supported home	106	7%
Lives with family	157	11%
Lives with partner	448	31%
Lives in residential home	112	8%
Missing	13	1%
Place of death		
Hospital	160	11%
Missing	1265	89%
Preferred place of death		
Home	144	10%
Hospice	53	4%
Hospital	114	8%
Nursing	2	0%
Not appropriate	287	20%
Declined	1	0%
Undecided	5	0%
Unable to choose	10	1%
Missing	305	21%
PPD achieved		
PPD achieved	N/A	N/A
PPD not achieved	N/A	N/A
Missing	N/A	N/A

Site 2 Community - Overall		
Variables	Number of observations	Average/Percentages
Total	937	100%
Age	937	72.30
Missing	0	0
Gender		
Female	448	48%
Male	489	52%
Missing	0	0
Ethnicity		
British	2	0%
White British	887	95%
White Irish	4	0%
White Scottish	1	0%
White	1	0%
Mixed British	13	1%
Black British	2	0%
English	8	1%
Welsh	1	0%
Greek	1	0%
Gypsy	1	0%
Italian	2	0%
Polish	2	0%
Baltic	1	0%
Iranian	1	0%
Muslim	1	0%
Hungarian	1	0%
Missing	7	1%
Religion		
Missing	937	100%
Interactions		
Weekend face to face consultations	142	1.04
Face to face consultations	794	2.85
Weekend telephone contacts	141	1.34
Telephone contacts	415	3.27
Weekend advice line	167	1.72
Advice line	265	2.20
Diagnosis		
Breast Cancer	56	6%
CNS Cancer	27	3%
Colorectal Cancer	84	9%
Headneck Cancer	59	6%
Lung Cancer	173	19%
Prostate Cancer	5	1%
Gastrointestinal Cancer	42	5%
Urological Cancer	28	3%

Site 2 Community - Overall		
Variables	Number of observations	Average/Percentages
Gynaeological Cancer	38	4%
Liver Cancer	18	2%
Skin Cancer	15	2%
Unknown Primary Cancer	13	1%
Pancreatic Cancer	87	9%
Haematological Cancer	2	0%
Renal Cancer	10	1%
Sarcoma Cancer	3	0%
Other Cancer	18	2%
COPD	28	3%
Respiratory	86	9%
Renal	6	1%
CVA	3	0%
Cardiac	0	0%
Heart Failure	21	2%
Dementia	14	2%
MND	6	1%
Parkinson	0	0%
Sclerosis	1	0%
Neurological Other	27	3%
Frailty	1	0%
HIV	0	0%
Haematological Other	27	3%
Liver Failure	3	0%
Other Diagnosis	20	2%
Missing	14	1%
Repetitions in data		
Number of times in dataset	937	3.18
Number of times in dataset on weekends	216	1.13
Cancer		
Has cancer	625	67%
Does not have cancer	312	33%
Missing	0	0%
AKPS	931	5278%
Missing	6	1%
Phase changes		
Phase changes observed	145	252%
No phase changes observed	187	591%
Pain		
No pain	203	22%
Slight pain	233	25%
Moderate pain	259	28%
Severe pain	186	20%
Overwhelm pain	41	4%
Missing	15	2%

Site 2 Community - Overall		
Variables	Number of observations	Average/Percentages
Weakness		
No weakness	57	6%
Slight weakness	151	16%
Moderate weakness	301	32%
Severe weakness	295	31%
Overwhelm weakness	115	12%
Missing	18	2%
Peace		
No peace	78	8%
Occasional peace	79	8%
Sometimes peace	128	14%
Most times peace	172	18%
Always peace	106	11%
Missing	374	40%
Social support		
Lives alone with help	162	30%
Lives alone with no help	14	3%
Lives in supported home	32	6%
Lives with family	146	27%
Lives with partner	191	24%
Missing	392	42%
Place of death		
Home	174	19%
Hospice	87	9%
Hospital	52	6%
Nursing	27	3%
Missing	597	64%
Preferred place of death		
Home	278	30%
Hospice	78	8%
Hospital	2	0%
Nursing	15	2%
Not appropriate	153	16%
Declined	47	5%
Undecided	57	6%
Unable to choose	4	0%
Missing	303	32%
PPD achieved		
PPD achieved	144	15%
PPD not achieved	229	24%
Missing	564	60%
